# Perinatal citalopram exposure alters the gut composition and microbial metabolic profiles of Sprague-Dawley rat dams and female offspring but not male offspring

**DOI:** 10.1186/s13293-025-00794-5

**Published:** 2025-12-03

**Authors:** Dawson R. Kropp, Matthew E. Glover, Rupabali Samanta, Keaton A. Unroe, Sarah M. Clinton, Georgia E. Hodes

**Affiliations:** 1 School of Neuroscience, Virginia Polytechnic Institute and State University, Integrated Life Sciences Building, 1981 Kraft Drive, Blacksburg, 24061 USA; 2 Fralin Life Sciences Institute, Virginia Polytechnic Institute and State University, Blacksburg, VA USA

**Keywords:** SSRI, Gut microbiome, Sprague-Dawley, Rat, Metagenomics, Shotgun sequencing, Next generation sequencing, Whole genome sequencing

## Abstract

**Background:**

Selective serotonin reuptake inhibitors are widely prescribed during pregnancy. Their main route of administration is through the gut. However, their impact on the maternal and offspring gut microbiome and microbial metabolic pathways remains poorly understood. This study used metagenomic shotgun sequencing to examine the effects of perinatal citalopram exposure in rat dams and their offspring on gut composition and downstream metabolic pathways.

**Methods:**

We treated pregnant and nursing rat dams with either citalopram or vehicle (water). Their feces were collected, DNA from these samples was extracted and then sequenced using shotgun metagenomic sequencing. The BioBakery suite of microbiome analysis tools was utilized in tandem with RStudio to analyze the gut composition and microbial metabolic pathways of the rat dams and their offspring.

**Results:**

Pregnant and nursing dams treated with citalopram exhibited marked shifts in microbial community structure, including phylum-level alterations in Proteobacteria and Defferibacteria. Citalopram treated dams displayed significantly altered beta diversity. Species level alterations due to treatment were composed of five significantly altered microbes, two of which belong to the Proteobacteria phylum. These changes were highly diverse and were not congruent with microbe-level alterations observed in offspring. Alpha diversity of microbial metabolic pathways was compared using the Gini-Simpson index, which was significantly increased in dams suggesting greater metabolic functional diversity with age. Female offspring perinatally exposed to citalopram showed significant changes in gut beta diversity, with seven significant alterations at the microbe level. These microbial shifts were accompanied by twenty-one significantly altered microbial metabolic pathways. In contrast, male offspring showed no significant differences in microbial composition or beta diversity and only minor metabolic changes.

**Conclusions:**

These findings demonstrate that maternal citalopram exposure during pregnancy and lactation has lasting, sex-specific impacts on the offspring’s gut microbiome and microbial metabolic pathways. The pronounced alterations in female, but not male offspring, suggest that host sex may be a critical determinant in the developmental response to citalopram exposure. This work underscores the value of metagenomic approaches in uncovering complex host-microbiome interactions and highlights the need to consider offspring sex in evaluating the safety and long-term effects of antidepressant use during pregnancy.

**Supplementary Information:**

The online version contains supplementary material available at 10.1186/s13293-025-00794-5.

## Abstract (plain language summary)

Selective serotonin reuptake inhibitors (SSRIs) like citalopram are commonly prescribed to pregnant women, yet their effects on the developing gut microbiome of offspring are not well understood. This study aimed to examine how citalopram exposure during pregnancy and nursing impacts gut bacteria and their metabolic influence both maternally and in offspring, with a focus on sex differences. Using cutting-edge metagenomic sequencing, we analyzed fecal samples from rat dams treated with citalopram, as well as from their male and female pups.

We found that citalopram exposure caused clear shifts in the gut microbiome and microbial metabolism in the dams and female offspring, but not in male offspring. Dams and female offspring showed changes in the diversity and composition of gut bacteria, as well as in the metabolic functions those microbes perform. Male offspring showed minimal changes, suggesting that they are less susceptible to SSRI-related microbial alterations.

Importantly, the gut microbial and metabolic profiles of female offspring resembled those of the mothers more closely than those of male offspring. These results highlight that exposure to SSRIs during development can lead to long-lasting and sex-specific changes in the gut microbiome.

Understanding how SSRIs impact the gut ecosystem is important for assessing the full spectrum of their effects during pregnancy and how that effects the subsequent generation. This research emphasizes the need to consider sex differences when evaluating the safety and long-term outcomes of antidepressant use during pregnancy.

**Supplementary Information:**

The online version contains supplementary material available at 10.1186/s13293-025-00794-5.

## Highlights

- Citalopram exposure during pregnancy and nursing alters gut microbiome composition in rat dams and female offspring but not male offspring.
- Significant changes were observed in microbial beta diversity and metabolic pathways in dams and female offspring only.
- Female offspring had reduced levels of Actinobacteria and showed altered metabolic functions, while male offspring remained largely unaffected.
- Gut microbial profiles in female offspring more closely resembled those of their mothers, indicating possible sex-dependent vertical transmission.
- Findings underscore the importance of sex as a biological variable when assessing the safety of SSRIs during pregnancy.

**Supplementary Information:**

The online version contains supplementary material available at 10.1186/s13293-025-00794-5.

## Background

Antidepressants are the most commonly prescribed class of drug for mood disorders, with 13.2% of people reporting having taken them in the last month [1, 2]. Among young adults aged 18–25, approximately two thirds of antidepressants prescribed were selective serotonin reuptake inhibitors (SSRIs), and they are twice as likely to be prescribed to women than men [2]. SSRIs are designed to prevent reuptake of serotonin by inhibiting serotonin transporter protein (SERT) [3, 4]. SSRIs are also commonly prescribed during pregnancy [5]. SSRIs can substantially improve the symptoms of individuals suffering from mood disorders, but it is important to understand the full implications of their use. Given that young women are more likely than men to be prescribed SSRIs, and that they are commonly prescribed to pregnant women, it is important to understand how SSRIs affect the mother and child throughout pregnancy and during the post partem period.

The most common route of administration for SSRIs is oral administration, therefore the gastrointestinal tract is a key player in the pharmacokinetics of SSRI absorption. SSRIs cause alterations in the gut microbiome of individuals taking them [6]. For instance, SSRIs such as fluoxetine and escitalopram have significant antimicrobial properties in the human gut [7]. Previous studies demonstrate that the SSRI fluoxetine can alter important gut microbial features in pregnant and lactating rat dams [8]. In human studies, the SSRI citalopram correlated with a significant increase to the family of gut microbes *Enterobacteriaceae* [9]. Therefore, it is important to understand how maternal treatment with SSRIs affects the gut microbiome of offspring.

Colonization of the gut microbiome at birth also presents a crucial window to influence the composition of the gut throughout life [10, 11]. This is important as it will set the stage for lifelong gut composition and critical immune system development. Vertical transmission, or transmission of microbes from parent to offspring, can be influenced by a variety of factors, including the method of delivery, breastfeeding, antibiotics, diet, and external environment [12–14]. It is vitally important to understand how SSRIs impact vertical transmission as this will have implications on the full life cycle of offspring and subsequent generations. Additionally, studies have found that there are sex differences in the colonization of the gut microbiome from mother to offspring [15]. Therefore, it is not only important to understand how SSRI exposure impacts offspring, but to understand how it does so sex specifically.

Despite the significant progress made in understanding the effects of maternal SSRI exposure on both dams and offspring, key gaps remain. Previous research is conflicting. Fluoxetine exposure did not alter the overall composition of the gut microbiome in C57BL maternal mice but did influence the transcriptomic profile of offspring brains [16]. However, in maternal rats with a history of early life stress, fluoxetine exposure did produce notable differences in gut microbiome composition [8]. It is unclear whether this is due to the impact of early life stress or the species differences. Stress exerts a greater influence on both maternal and neonatal microbiota than fluoxetine alone suggesting that the mother’s previous life experience contributed to the disparities [17]. In utero fluoxetine exposure has been associated with decreased alpha diversity and alterations in key bacterial genera, including *Turicibacter*, *Parasutterella*, and *Romboutsia* in unstressed mice [18]. However, to date, studies have used 16S rRNA sequencing to address this question with a focus on the SSRI fluoxetine. While these studies provide valuable insights, more information is needed regarding the in-depth effects of early-life SSRI exposure on the gut. Our study addresses this gap by determining how the SSRI citalopram influences the gut microbiome composition and microbial metabolic pathways of rats using metagenomic shotgun sequencing. Shotgun sequencing serves as a more comprehensive approach than traditional amplicon sequencing. This method enables a broader characterization of microbial communities and provides deeper insights into the downstream metabolic consequences of in utero SSRI exposure. The SSRI we have chosen for this investigation is citalopram as it is one of the most widely prescribed SSRIs during pregnancy and can pass through breast milk [19]. Offspring are also exposed to the drug in utero as SSRIs are capable of passing through the placenta [20]. As such citalopram can influence offspring microbiome development *in utero* and through weaning. Citalopram safety has been previously assessed and can be prescribed during pregnancy, despite its potential to impact microbial transmission in offspring [21, 22].

We performed experiments aimed at addressing the question of how perinatal exposure to the SSRI citalopram sex specifically impacts the developing gut microbiome of rats. We treated adult female Sprague Dawley rats with citalopram during breeding, pregnancy, and breast feeding. We evaluated the effects of SSRI exposure on the gut microbial environment of both dams and their offspring. Using metagenomic shotgun sequencing to analyze fecal samples, we assessed microbial composition at key developmental time points, investigating potential sex-based differences in microbial colonization and downstream metabolic pathways. These findings contribute to our understanding of the broader implications of SSRI use during pregnancy on gut microbiome dynamics across generations and sex in rats.

## Methods

### Animals

#### Dams

Male and female breeder rats were purchased from Charles River Laboratories (Kingston, NY, USA). Animals were housed in a temperature- and humidity-controlled facility (21–23 °C, 50%-55% humidity) with 12/12 h light–dark cycle (lights on 0700); food and water were available ad libitum. Adult female Sprague Dawley rats (*n* = 16) were paired-housed with male breeders to generate offspring. Evidence of successful mating was checked daily in the first two hours of the light cycle by vaginal lavage and visual evaluation for a sperm plug. Male breeders were removed once mating was confirmed. All procedures were performed in accordance with the Institutional Animal Care and Use Committee guidelines of Virginia Tech.

### Offspring

At birth, litters were culled to six male/six female pups. To control for litter effects, no more than two pups per litter were used for later study. Pups remained with dams undisturbed except for routine cage changes, removal of dams for weighing, and replacement/refilling of water bottles until weaning on P21. At weaning, male (*n* = 20) and female (*n* = 20) offspring from citalopram and vehicle treated mothers were group housed in separate cages to avoid microbial contamination between groups (*n* = 3 per cage). All animals were housed with paper bedding and maintained on a 12-h light/dark cycle with ad libitum access to food and water. All procedures were performed in accordance with the Institutional Animal Care and Use Committee guidelines of Virginia Tech.

#### Citalopram and vehicle administration

Adult female Sprague Dawley rats randomly received either citalopram dissolved in drinking water (10 mg/kg/day); or vehicle (tap water) for a week prior to and through mating, as well as pregnancy and the postpartum period. This dosage and timeline were selected as it has been shown to cause observable developmental alterations to offspring exposed during early life [23–27]. The citalopram solutions were replaced every two days, with concentrations adjusted based on each rat’s body weight and average water consumption, determined by weighing the drinking bottles at the time of replacement.

#### Fecal sample collection

**Fig. 1:**
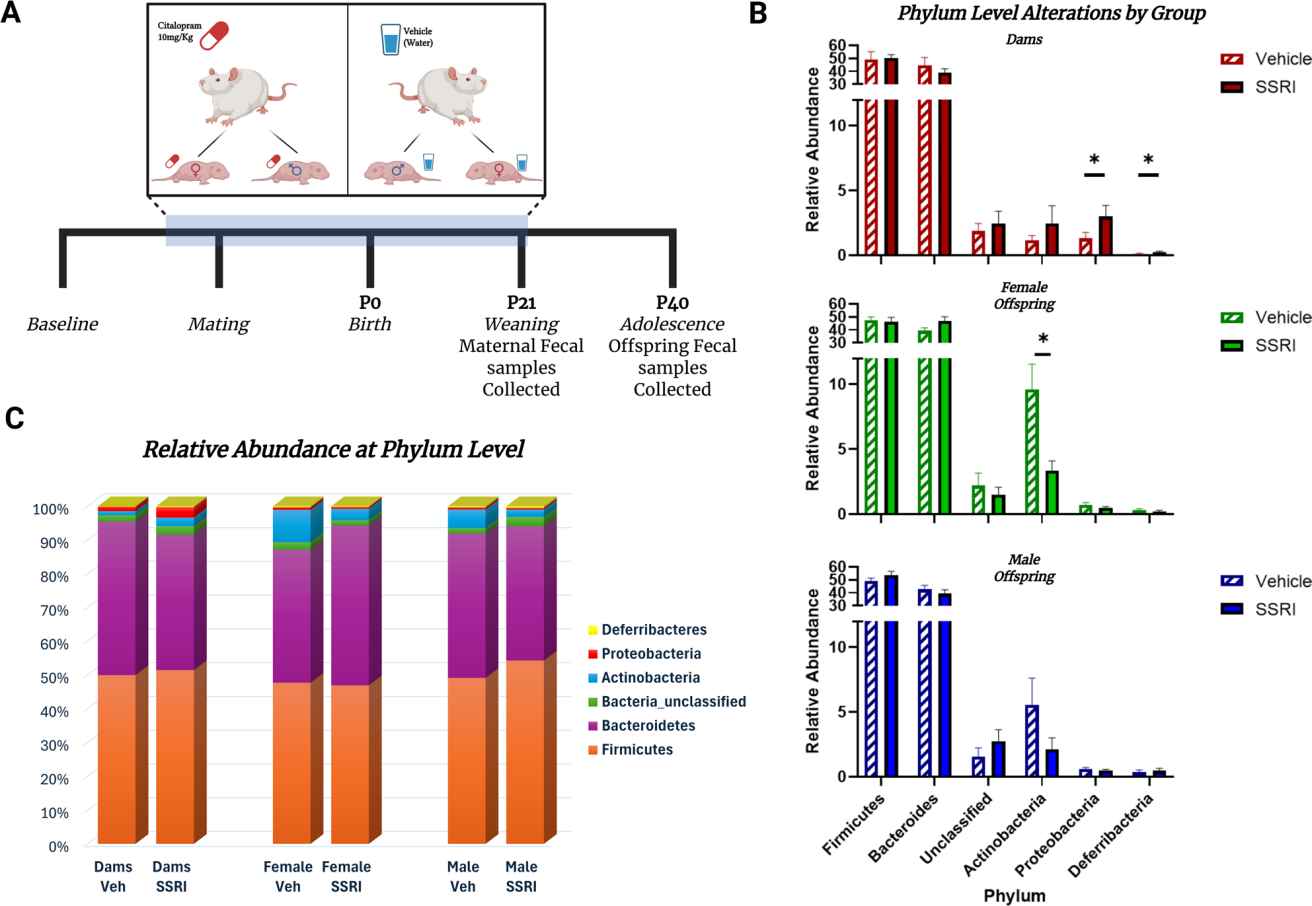
Schematic of citalopram or vehicle administration in dams and resulting effects on offspring groups (**A**). Bar graphs represent the relative abundance of each phylum with a Mann-Whitney U test performed for each phylum by group, treatment vs. vehicle represented mean ± SEM (* = q < 0.25) (**B**). Bar graph showing the relative abundance of the detected bacterial phyla across all treatments and groups (**C**)

On P21, fecal samples were collected from dams in order to observe the gut microbiome at time of weaning. In order to study the gut microbiome of offspring at adolescence, fecal samples were collected (*n* = 14–20/condition/sex) from male and female offspring on P40 (Fig. 1A). One fecal sample was collected per cage for analysis and immediately frozen at -80 °C for processing and analysis. This method has previously been employed by our lab and has been shown to generate detectable levels of drug in both blood and brain of offspring [28].

#### Next generation shotgun sequencing

Fecal samples were collected from dams and offspring for sequencing analysis. DNA from the samples was isolated using the Quick-DNA™ Fecal/Soil isolation kit (Zymo Research). Extracted DNA was sent to the Fralin Life Science Institute Genomics Research Laboratory (GRL) at the Virginia Tech Biocomplexity Institute where they prepared DNA libraries and performed next generation shotgun metagenomic sequencing. Samples were sequencing using an Illumina Novaseq 6000 using the S1 flow cell. This flow cell produced 12 million paired end reads at 100 base-pair length each.

#### Sequencing analysis

Quality controlled fastq files were generated by the Genomic Research Laboratory at Virginia Tech and sent to our lab. In order to remove host genome contamination, fastq files were aligned to the Sprague Dawley reference genome GRCr8 (GCF_036323735.1) using Bowtie2 [29]. Matches to the host genome were removed. Filtered and host removed sequences were then run through Metaphlan 4.0 which outputs relative abundance of various taxa based on database marker genes [30]. These filtered and host removed fastq files were then run through the program HUMAnN v3.8 using Uniref50 to map microbes in order to determine functional activity of microbes via defined metabolic pathways [31]. Differential abundance analysis between treatment groups and generational factors was performed using Maaslin2 with a default significance threshold of q-val < 0.25 [32]. PCoA plots were plotted using bray-Curtis distances, ellipses around the groups represent a 95% confidence interval. PERMANOVA analysis was run on the same bray-Curtis distances.

#### Statistical analysis

Microbial relative abundance data were analyzed in GraphPad Prism (Version 10.2.3, GraphPad, La Jolla California USA). Mann-Whitney U tests corrected for multiple comparisons were performed to determine significant differences in phyla between experimental groups. Significance thresholds for the Mann-Whitney U tests were set at q < 0.25. A Welch’s t-test was performed to compare the metabolic alpha diversity between all offspring and dams. One-way ANOVA was utilized to find significant variance in metabolic alpha diversity levels between dams, male offspring, and female offspring. Tukey’s multiple comparison test was performed to determine significant differences between each of the three groups.

## Results

### Phylum level alterations occur in dams and female offspring after citalopram exposure, but not in male offspring

Rat dams were treated with either citalopram or vehicle (water) during mating, pregnancy, and weaning (Fig. 1A). We used a Mann-Whitney U test corrected for multiple comparisons to identify significant differences between the SSRI and vehicle exposed gut composition at the phylum level by group. In dams we found significant differences in Proteobacteria (q = 0.12) and Defferibacteria (q = 0.12) (Fig. 1B). In female offspring there was a significant decrease in the relative abundance of Actinobacteria (q = 0.089) (Fig. 1B). Males displayed no significant differences at the phylum level due to SSRI or Vehicle exposure (Fig. 1B). Relative abundance of gut bacteria was quantified and graphed at the phylum level by experimental group (Fig. 1C). Together these data demonstrate that citalopram is capable of causing compositional changes at the phylum level in female offspring and dams, but not in male offspring. Although there were significant changes at the phylum level in dams, it was not the same phyla that changed. This suggests that treatment exposure in female offspring has an effect, but that it is not congruent between dams and offspring. Relative abundance of bacteria at each taxonomic level can be found in the supplemental materials (S2).

### Treatment exposure and animal generation drive changes to gut composition

**Fig. 2:**
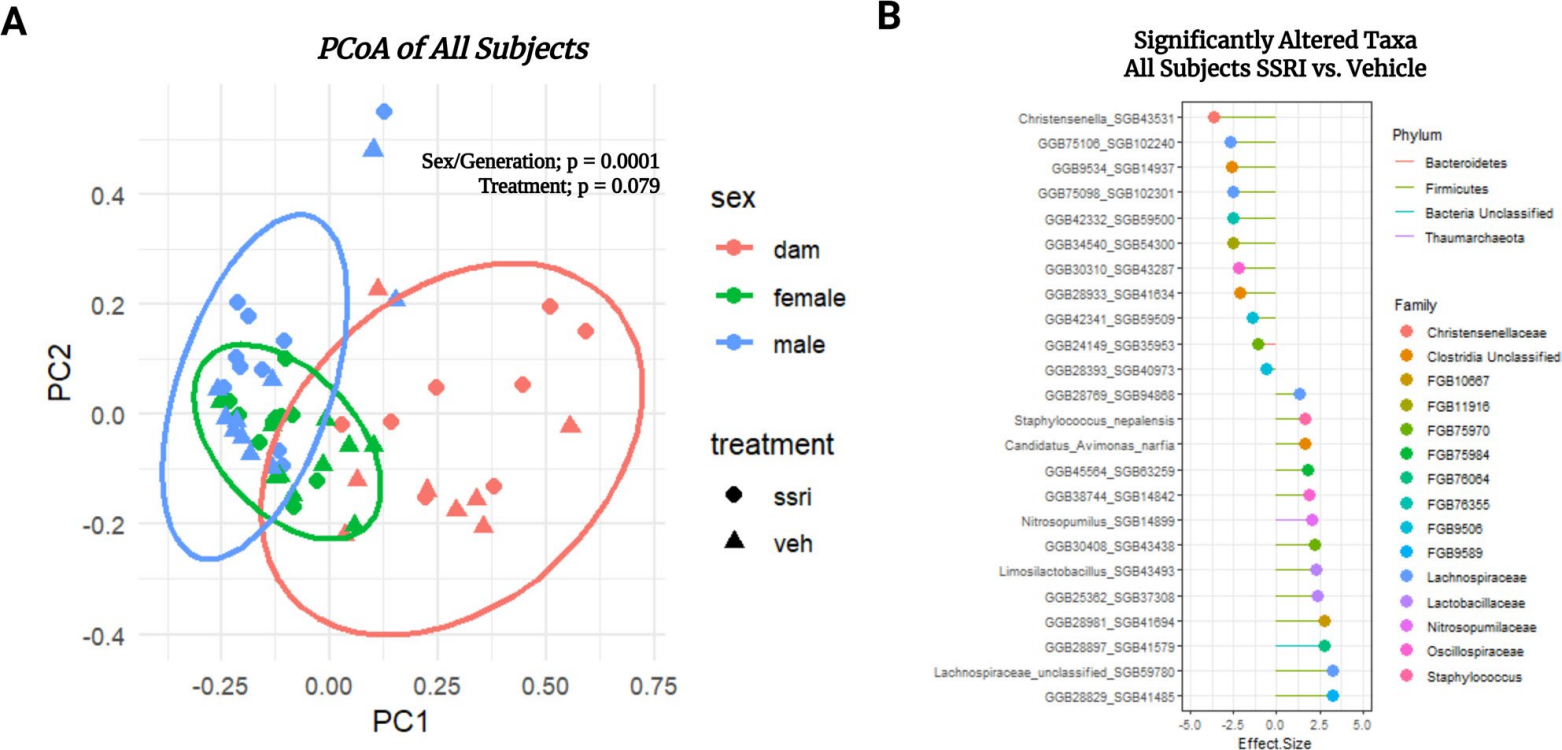
PCoA of bray-curtis distances in all subjects and all treatment groups. Females are represented in green, males are represented in blue, and dams are represented in red; circles represent control subjects and triangles represent SSRI treated subjects. Ellipses display a 95% confidence interval, p-values generated from PERMANOVA comparing treatment to vehicle, and group membership (male offspring v female offspring v dams) (**A**). Dot plot showing the effect size of significantly altered species comparing all SSRI treated/exposed (*n* = 28) to all vehicle treated/exposed (*n* = 28). Species of the microbes altered are listed on the y-axis, their family membership is represented as the dot color, and the phylum membership is represented as the line segment color (**B**)

We sought to understand how treatment exposure and generation affected beta diversity of the gut composition of all subjects. We did so by calculating Bray-Curtis distances, then running PERMANOVA analysis to determine significant differences. PERMANOVA revealed there was a significant difference between all offspring and maternal rat beta diversity regardless of treatment (F = 8.67, Df = 2, *p* = 0.0001), but not a significant difference due to treatment alone when considering all animals (F = 1.683998342, Df = 1, *p* = 0.0792) (Fig. 2A). Analysis of significant differences at the species level due to treatment exposure was performed in all subjects using Maaslin2. This analysis revealed 24 significantly altered microbe species between citalopram exposed rats compared to vehicle exposed. These significantly altered microbes spanned across 15 families and 4 phyla (Fig. 2B). Although the effect of treatment is insignificant, the very significant effect of sex/generation raised the question of whether within-group differences were being obscured when they were collapsed together. Furthermore, there is a significant interaction between treatment status and group identity (F = 2.03, Df = 2, *p* = 0.01). We therefore sought to analyze the data within group. PERMANOVA results for all comparisons can be found in the supplemental materials (S3).

### Beta diversity in dams and female offspring is affected by treatment exposure, in male offspring it is not

**Fig. 3:**
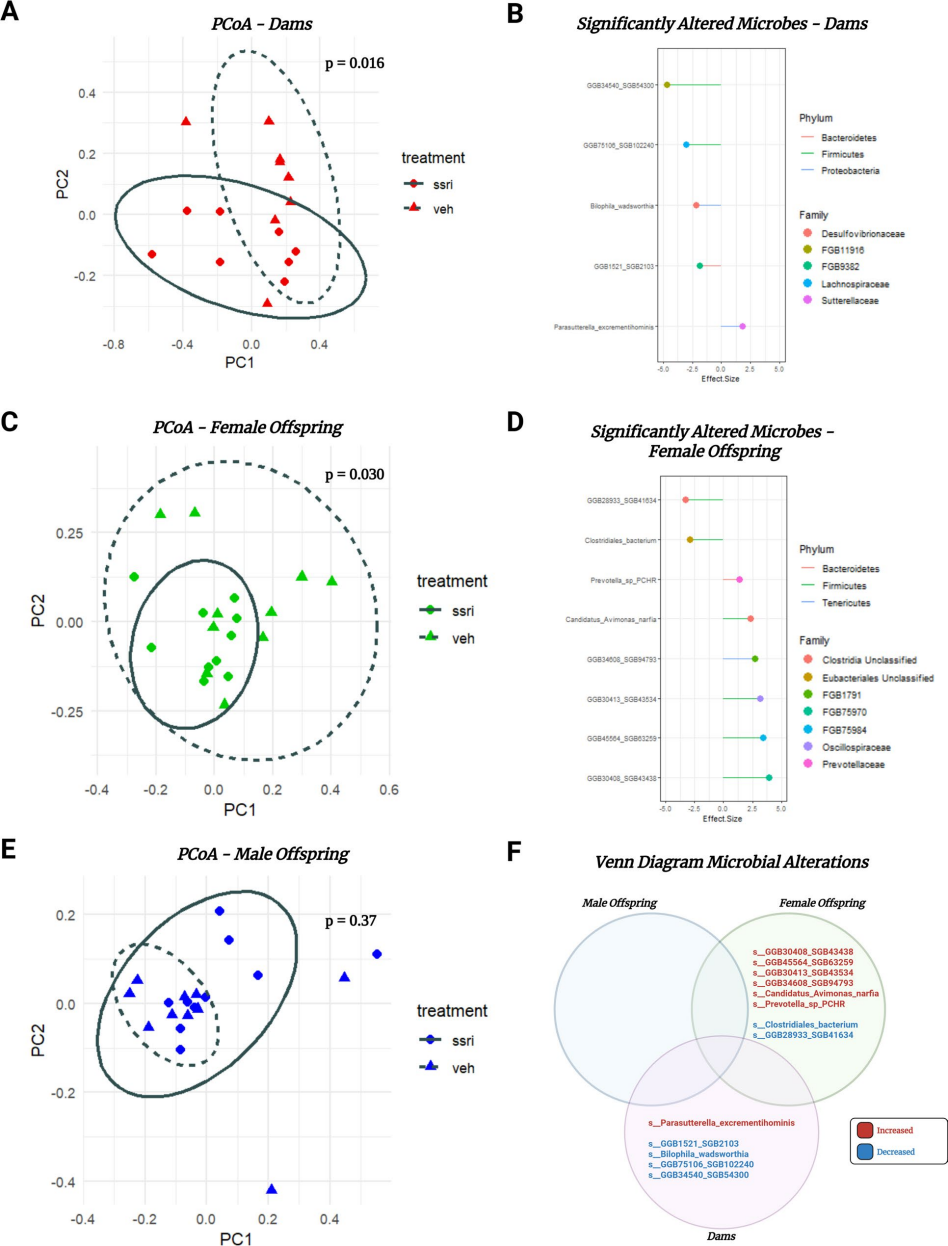
PCoA of bray-curtis distances in dams only with p-values determined with PERMANOVA analysis in treatment vs. vehicle. Red circles and solid ellipses represent SSRI treated dams and red triangles with dotted ellipses represent vehicle treated dams (**A**). Dot plot showing the effect size of significantly altered species of significantly altered species exclusively in the dams group. Species of the microbes altered are listed on the y-axis, their family membership is represented as the dot color, and the phylum membership is represented as the line segment color (**B**). PCoA of bray-curtis distances in female offspring only with p-values determined with PERMANOVA analysis in treatment vs. vehicle. Green circles and solid ellipses represent SSRI exposed female offspring and green triangles with dotted ellipses represent vehicle exposed female offspring (**C**). Dot plot showing the effect size of significantly altered species of significantly altered species exclusively in the female offspring group (**D**). PCoA of bray-curtis distances in male offspring only with p-values determined with PERMANOVA analysis in treatment vs. vehicle. Blue circles and solid ellipses represent SSRI exposed male offspring and blue triangles with dotted ellipses represent vehicle exposed male offspring (**E**). Venn diagram showing the overlap of significantly altered species between dams and offspring due to treatment, red text represents a significant increase in relative abundance in the SSRI group and blue text a significant decrease (**F**)

Within-group comparisons were performed utilizing PCoA and PERMANOVA in citalopram treated or exposed vs. vehicle for dams, male offspring, and female offspring groups individually. PERMANOVA revealed that beta diversity between treated and vehicle dams demonstrates significant differences (F = 2.38, Df = 1, *p* = 0.016) (Fig. 3A). Species comparisons using Maaslin2 revealed that dams had 5 significantly altered species belonging to 5 unique families and 3 unique phyla (Fig. 3B). PERMANOVA reveals that female offspring exposed to citalopram treatment or vehicle also had significant differences in beta diversity (F = 1.94, Df = 1, *p* = 0.030) (Fig. 3C). Species comparisons using Maaslin2.0 revealed that female offspring treated with citalopram had 8 significantly altered species, these species belonged to 7 unique families and 3 unique phyla (Fig. 3D). Full results tables comparing microbes across treatment groups are available in the supplemental materials (S4). PERMANOVA of the male offspring rats demonstrated no significant differences between treatment and vehicle groups (Fig. 3E). There was no overlap in the microbes significantly altered in the dams with those significantly altered in the male and the female offspring (Fig. 3F). This again demonstrates that female offspring are significantly affected by SSRI exposure but not congruent with alterations seen in dams. PERMANOVA results for all comparisons can be found in the supplemental materials (S3).

### Metabolic pathways display more diversity in dams compared to offspring

**Fig. 4:**
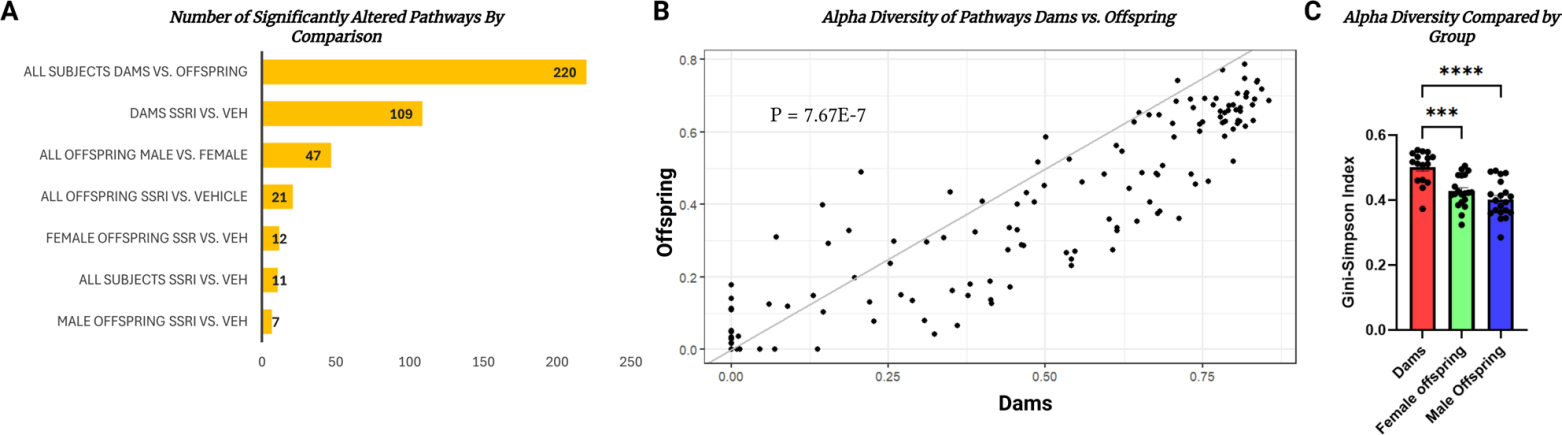
Summary bar graph showing the number of significantly different microbial metabolic pathways from humannv3.8 using maaslin2 in a given different variables and groups. Comparisons were on the axes of generation, sex, or treatment status and contained variable numbers of subjects based on group membership (**A**). Alpha-diversity scatterplot comparing diversity of proteins in stratified pathways comparing dams and offspring rats. P-value determined with an unpaired t-test between the mean Gini-Simpson index of all dams vs. all offspring (**B**). Bar graph plotting the mean Gini-Simpson index in each group, one-way ANOVA and tukeys multiple comparison tests were used to compare groups for significant differences (*** = p-adj < 0.001; **** = p-adj < 0.0001) (**C**)

In addition to compositional changes, we sought to systematically compare the effect of sex, generation, and treatment on microbial metabolic pathways. We utilized HUMAnN v3.8 in tandem with the Uniref50 and MetaCyc databases in order to characterize metabolic pathways. We then used Maaslin2 to compare the abundance of those pathways. A comparison of generation regardless of treatment group (all dams vs. all offspring) revealed 220 significantly altered functional pathways. This comparison based on generation displayed the greatest number of altered metabolic pathways. The comparison that yielded the second most differences was in dams only, SSRI vs. vehicle treated, with 109 altered pathways. Together these results demonstrate that dams and offspring have distinct microbial metabolic profiles, and that the profile from dams shows greater alterations from treatment. The next most impactful variable was the comparison of sex in offspring alone collapsing the treatment conditions (all male offspring vs. all female offspring), with 47 differential pathways. This shows that the sex condition is more impactful for offspring than the treatment condition alone (Fig. 4A).

Exposure to treatment resulted in far fewer metabolic pathway alterations for offspring. In the comparison of treatment vs. vehicle exposed in all offspring regardless of sex, there were 21 metabolic pathway differences. When including only female offspring, treatment condition accounted for 12 differential pathways. When comparing treatment conditions and including all subjects, we found 11 differential pathways. Finally, when comparing treatment conditions in just male offspring, we found the fewest altered pathways with 7 differences (Fig. 4A).

The functional differences between dams and offspring regardless of treatment implied an increased functional diversity over time. A Welch’s t-test comparing the alpha diversity of metabolic pathway using a Gini-Simpson index revealed significant differences between dams and offspring (*p* = 7.37E-7) (Fig. 4B). We sought to understand if any group in particular was driving this effect. A one-way ANOVA revealed significant variance between groups (F = 17.26, Df = 2, *p* < 0.0001). Tukey’s multiple comparison test revealed significant differences between dams and male offspring (p-adj < 0.0001), dams and female offspring (p-adj = 0.0002), but there was no difference between male and female offspring on mean alpha diversity (p-adj = 0.27) (Fig. 4C). Together these data demonstrate that the dams were most affected metabolically due to treatment and that they had a greater diversity of proteins comprising their metabolic pathways. Treatment/exposure had no effect on metabolic alpha diversity in offspring. While both males and female offspring differed from dams, males had more differences than female offspring. A list of each subject and the mean alpha diversity of their metabolic pathways can be found in the supplemental materials (S5).

### Metabolic pathway alterations that occur due to SSRI exposure are dominated by the dams group

**Fig. 5:**
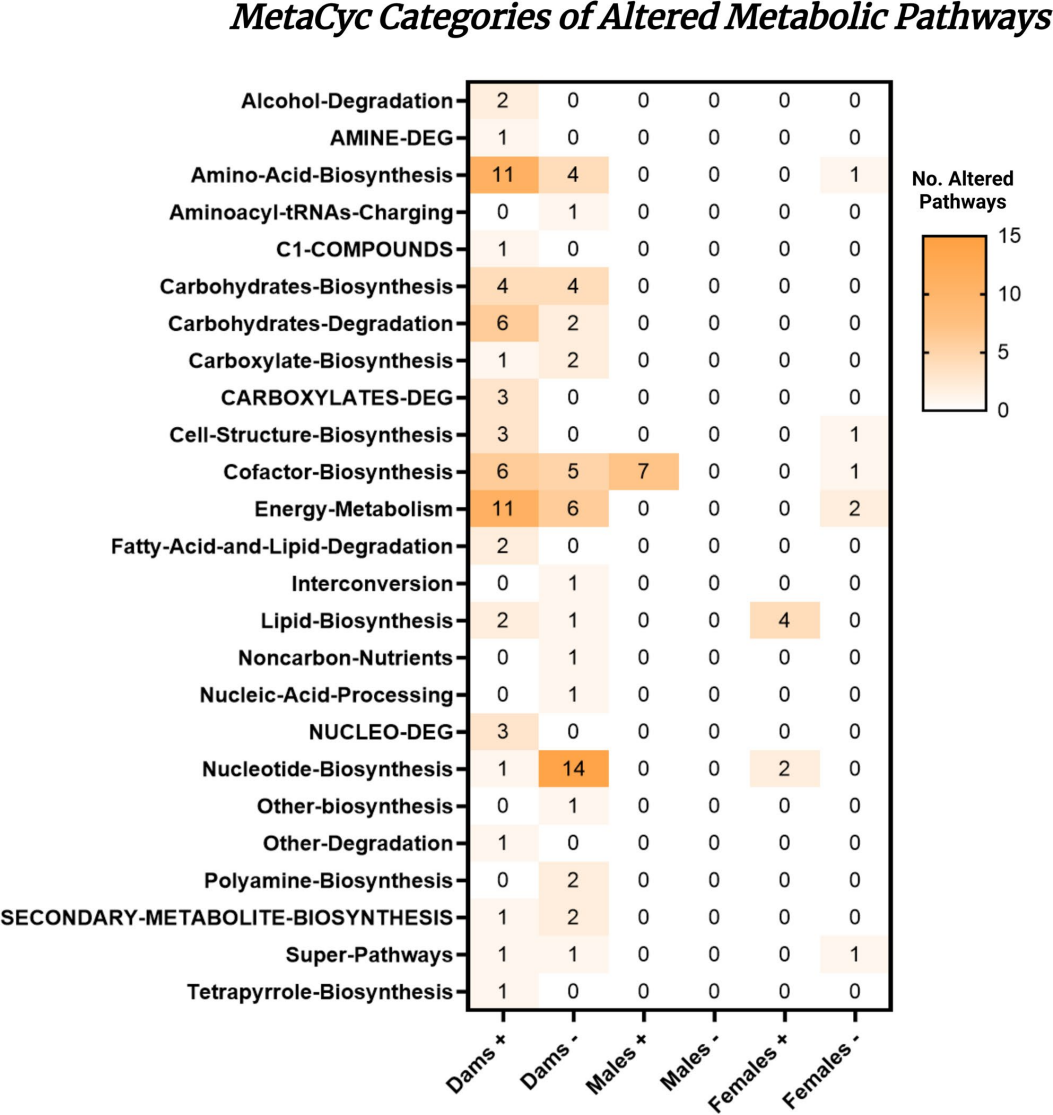
Summary of microbial metabolic pathway analysis categorized by orthological group. Comparisons were within-group (dams SSRI vs. dams vehicle; male offspring SSRI vs. male offspring vehicle; female offspring SSRI vs. female offspring vehicle). (+) denotes pathways that were significantly increased, (-) denotes pathways that were significantly decreased. Shades of yellow represent how many pathways were significantly altered in each category with darker shades representing more pathways

In order to understand these microbial metabolic changes from a functional perspective, an orthological analysis of the sequencing data was performed using the MetaCyc database. Results from the comparison of treatment vs. vehicle in dams, female offspring, and male offspring were grouped based on MetaCyc categories. Citalopram treated Dams had more significantly altered metabolic pathways than that of their offspring (Fig. 5). The most abundantly increased metabolic pathways measured in SSRI exposed dams had to do with energy metabolism, amino acid biosynthesis, cofactor biosynthesis, and carbohydrate degradation (Fig. 5). Most commonly decreased metabolic pathways in SSRI treated dams were nucleotide biosynthesis, energy metabolism, and cofactor biosynthesis (Fig. 5). Male offspring of dams treated with citalopram only had one category of altered pathways, which was an increase in cofactor biosynthesis (Fig. 5). Deeper analysis reveals that all of these pathways are related to the biosynthesis of menaquinol, most likely representing a functional overlap with other menaquinol pathways. A full list of pathways comparisons by group can be found in the supplemental materials (S6). Female offspring of citalopram treated dams saw an increase in pathways related to lipid biosynthesis and nucleotide biosynthesis. These females had a decrease in energy metabolism, cell structure biosynthesis, cofactor biosynthesis, and super pathways (Fig. 3A). Full lists of which pathways fell into what MetaCyc categories can be found in supplementary materials (S7).

## Discussion

This study provides a novel perspective into the effects of perinatal citalopram exposure in rat dams and offspring using metagenomic shotgun sequencing. Our findings contribute to the growing body of literature on the impact of SSRI treatment during pregnancy. Previous studies employing 16S rRNA amplicon sequencing have demonstrated that SSRI use alters the maternal metabolome but not the gut composition [8, 16]. However, no studies have done so using metagenomic tools and therefore may miss important data, especially involving microbial metabolic activity. Our study demonstrated that in pregnant and weaning dams, SSRI treatment leads to significant alterations in the phyla Proteobacteria and Defferibacteria. Beta diversity of the gut composition in dams shows significant differences, driven by five significantly altered microbes. In female offspring there are also significant differences in beta diversity due to treatment exposure in utero and weaning. This change in beta diversity was driven by seven altered microbes. Interestingly, males had no significantly altered microbes and their beta diversity showed no difference in treatment exposed vs. vehicle exposed. At the microbe metabolic pathway level dams had many pathways significantly altered due to treatment with one-hundred nine pathways altered. Offspring only demonstrated twenty one altered pathways due to treatment exposure. An alpha diversity analysis using Gini-Simpson of diversity of proteins within metabolic pathways showed that dams had significantly more diversity within each metabolic pathway.

### Gut composition of female offspring shows greater similarity to dams compared to the gut composition of male offspring

In performing PCoA and analysis of Bray-Curtis distances, we found that the gut composition of female offspring shows more similarity to dams than that of male offspring. This may explain why female offspring demonstrated altered gut microbes and male offspring did not. Despite these similarities there was still no overlap in dams and female offspring for significantly altered species or phyla due to treatment. This may be due to the different routes of exposure, where dams had the drug directly pass through their gut, whereas offspring would have been exposed through the placenta and lactation. Therefore, these differences between mother and offspring may be the result of primary vs. secondary exposure. More studies would need to be done parsing out the exact mechanism driving these discordant gut alterations.

### Perinatal citalopram exposure decreases the relative abundance of Actinobacteria in female offspring but not in male offspring

Despite a small relative abundance compared to phyla such as Firmicutes and Bacteroides, Actinobacteria play a key role in gut regulation [33]. In this study we found that female offspring had decreased relative abundance of the phylum actinobacteria compared to unexposed female offspring. In male offspring a decreasing trend was seen with Actinobacteria but was not significant, leading us to conclude that this effect is exclusive to females. A previous study in human subjects demonstrated that increased levels of Actinobacteria were associated with a diagnosis of MDD [34]. This may represent a potential antidepressant mechanism being passed onto female offspring through SSRI exposure. More studies are required to assess if this decrease in actinobacteria is associated with less anxiety-like and depression-like outcomes in rats.

### Compositional alterations are revealed when looking at the data sex specifically and by generation

When measuring the gut composition of all animals there were no significant differences in beta diversity (Bray-Curtis distances) due to treatment, but many significant species-level alterations. When we performed our analysis sex-specifically it revealed alterations that had not previously been identified. The most important results demonstrated that female offspring and dams had their beta diversity significantly altered by treatment or exposure, but that male offspring did not. Interestingly, microbes from the phylum Firmicutes were only downregulated in treated dams, but both upregulated and downregulated in exposed female offspring (Fig. 3B and D). What is seen in female offspring here is consistent with previous mouse work from our group that demonstrated both upregulated and downregulated Firmicutes in stressed female mice [35]. This suggests Firmicutes play a dynamic role in the regulation of psychological and physical stress. Caution should be taken to not generalize Firmicutes into an absolute category as entirely good or bad for depression outcomes, instead it should be assessed on a species-by-species basis. Additionally, in our mouse study we found a decrease of a microbe in the *Lachnospiraceae* family, which also occurred following SSRI treatment in rat dams (Fig. 3B). This may demonstrate a mechanism for protection against stress-like conditions.

### Several altered microbial metabolic pathways have implications on MDD mechanisms in literature

In order to contextualize these results, we looked into the literature to find connections to other insights into maternal gut composition with SSRI treatment. Previous studies have demonstrated with 16S rRNA amplicon sequencing that maternal fluoxetine exposure has been linked to shifts in the genus *Parasutterella* and reduced offspring alpha diversity [18]. Our results align with this; we found that dams had an increased relative abundance of *Parasutterella* driven by the species *Parasutterella excrementihomis*. However, we did not observe alterations in the genera *Turicibacter* and *Romboutsia*, which may be attributable to differences in SSRI type (fluoxetine vs. citalopram), sequencing methodologies (amplicon vs. shotgun), or species-specific effects (mouse vs. rat). Further studies utilizing a range of SSRIs, diverse host species, and metagenomic sequencing are essential to deepen our understanding of the gut microbiome’s response to perinatal SSRI exposure.

In addition to compositional changes, we also sought to contextualize some of the microbial metabolic changes we observed with established literature. In this study we found that Inosine-5’-phosphate is decreased in citalopram treated dams and is unaltered between exposed and vehicle offspring (S6). This finding is relevant as lower inosine is associated with depression in human children as well as adults [36, 37]. Additionally, we found a significant decrease in the de novo biosynthesis of NAD, which is the end-product of the kynurenic acid tryptophan pathway (S6). A decrease in NAD production is consistent with previous findings where more demand is put on the serotonin side of the tryptophan metabolic pathway, leading to less activation of the kynurenic acid pathway during SSRI treatment [38, 39]. L-serine and glycine biosynthesis were also significantly decreased in the SSRI treated dams (S6). In humans, significantly lower plasma glycine values were associated with depression [40]. This may suggest a deleterious mechanism of early-life SSRI exposure; more studies would need to be performed to investigate whether this leads to a depression-like phenotype behaviorally. There are some conflicting findings with this however where metabolism of serine and glycine was found to increase in depressed patients [41]. L-arginine biosynthesis significantly increased in citalopram treated dams (S6). L-arginine has been shown to decrease in MDD, suggesting a potential protective mechanism for SSRIs [42].

### Rat dams display far greater metabolic alterations due to citalopram treatment compared to exposed offspring

The treated vs. vehicle dams displayed the most functional differences with 109 significantly altered microbial metabolic pathways due to treatment, offspring generally or sex-specifically demonstrated substantially fewer altered pathways (Fig. 4A). These data suggest that the metabolic alterations due to citalopram exposure are confined mostly to the treated dams, and not the exposed offspring. In addition to this, there were more differences in functional pathways identified by comparing sex or generation than that of citalopram exposure in the offspring (Fig. 4A). This may be due to the fact that dams simply have more diverse metabolic pathways due to specialization over time. This is supported by the increased alpha diversity displayed in the metabolic pathways of the dam group (Fig. 4B and C). However, there is a chance that this effect is due to the differing routes of administration (direct treatment vs. exposure). Additionally, previous studies have demonstrated that SSRI treatment in rodents perinatally resulted in reduced fecal amino acid concentrations for dams [16]. This could also be a mechanism by which sex differences in gut composition changes are observed. Pathways for amino acid biosynthesis were significantly altered in both dams and female offspring in our study, but not in male offspring (Fig. 5). More studies examining these differences in microbial metabolic profiles in parents versus offspring would be beneficial for understanding the dynamics of vertical transmission of gut microbes.

### Limitations

In order to contextualize these findings, we must address the limitations of the current study. This study sought to understand the effect of citalopram exposure on gut microbiome composition and metabolomic activity. This study does not contain behavioral data or metabolite data (MS/LC-MS). Future studies integrating these findings with behavioral data and metabolites would help to strengthen the conclusions made here. Another limitation is that this study is limited by the current state of microbial databases. Many microbes have yet to be identified and described, therefore we may make assumptions about higher level taxonomic information (i.e. phylum membership), however as these databases become updated it may be useful to re-address the data presented here with new microbial insights. One limitation of Metaphlan-related tools is the inability to assess alpha diversity of gut composition. This is due to the fact that Metaphlan does not produce count data, and therefore alpha diversity cannot be accurately calculated. Additionally, with this study we could not parse out the effect of placental citalopram exposure vs. exposure from breast milk. Future studies parsing these two periods of exposure could glean new light into the mechanisms underlying microbial vertical transmission. Finally, handling of dams for breeding and citalopram treatment could possibly be a stressor. More studies would need to be done with the absence of human handling to understand if any of these effects are stress related.

## Conclusion

Our study sought to address how citalopram treatment in rat dams affected the gut microbiome and microbial metabolic pathways of offspring using metagenomic shotgun sequencing. Male offspring appear mostly unaffected by citalopram exposure in utero and weaning, with no compositional alterations and very minor microbial metabolic changes. Female offspring however showed significant compositional changes at the level of individual microbes as well as beta diversity generally. Metabolically, female offspring demonstrated some changes at the pathway level but not close to the number altered in dams. Dams displayed significant compositional changes at the microbe level and phylum level. They also demonstrated the most metabolic changes due to treatment, most likely driven by a higher level of metabolic specialization. These data show that treatment of pregnant dams with citalopram in utero in weaning has a significant effect the gut microbiome of female offspring, but not male offspring. This implies that the sex of the offspring should be a consideration for the safety of SSRI treatment in pregnant women. Specifically, female offspring may be more at risk of gut microbiome alterations due to SSRI exposure compared to male offspring. These gut alterations may be beneficial or deleterious but will have long-term consequences for offspring development.

## Supplementary Information

Below is the link to the electronic supplementary material.

Supplementary Material 1: Full tables of statistics from Mann-Whitney U test comparisons at the phylum level in each group.

Supplementary Material S2: Relative abundance of each microbe at all taxonomic levels in all subjects.

Supplementary Material S3: Full tables of statistics from PERMANOVA comparisons in all subjects and by group.

Supplementary Material S4: Full results tables for maaslin2 comparisons at the species level in all subjects and by group.

Supplementary Material S5: List of the mean Gini-Simpson index for the metabolic pathways of each subject.

Supplementary Material S6: Full results tables for maaslin2 comparisons of metabolic pathways by group and variable compared.

Supplementary Material S7: Tables of metabolic pathways and their orthological class based on the MetaCyc database.

## Data Availability

Data from the findings in this paper can be found in the supplementary materials. Any data not available here is available upon request to the corresponding author.
